# GalR, GalX and AraR co‐regulate d‐galactose and l‐arabinose utilization in *Aspergillus nidulans*

**DOI:** 10.1111/1751-7915.14025

**Published:** 2022-02-25

**Authors:** Jiali Meng, Zoltán Németh, Mao Peng, Erzsébet Fekete, Sandra Garrigues, Anna Lipzen, Vivian Ng, Emily Savage, Yu Zhang, Igor V. Grigoriev, Miia R. Mäkelä, Levente Karaffa, Ronald P. de Vries

**Affiliations:** ^1^ Fungal Physiology Westerdijk Fungal Biodiversity Institute & Fungal Molecular Physiology Utrecht University Uppsalalaan 8 Utrecht 3584 CT The Netherlands; ^2^ 37599 Department of Biochemical Engineering Faculty of Science and Technology University of Debrecen Egyetem tér 1. Debrecen H‐4032 Hungary; ^3^ Lawrence Berkeley National Laboratory US Department of Energy Joint Genome Institute 1 Cyclotron Road Berkeley CA 94720 USA; ^4^ Department of Microbiology University of Helsinki Viikinkaari 9 Helsinki 00790 Finland; ^5^ 37599 Institute of Metagenomics University of Debrecen Egyetem tér 1. Debrecen H‐4032 Hungary

## Abstract

Filamentous fungi produce a wide variety of enzymes in order to efficiently degrade plant cell wall polysaccharides. The production of these enzymes is controlled by transcriptional regulators, which also control the catabolic pathways that convert the released monosaccharides. Two transcriptional regulators, GalX and GalR, control d‐galactose utilization in the model filamentous fungus *Aspergillus nidulans*, while the arabinanolytic regulator AraR regulates l‐arabinose catabolism. d‐Galactose and l‐arabinose are commonly found together in polysaccharides, such as arabinogalactan, xylan and rhamnogalacturonan I. Therefore, the catabolic pathways that convert d‐galactose and l‐arabinose are often also likely to be active simultaneously. In this study, we investigated the interaction between GalX, GalR and AraR in d‐galactose and l‐arabinose catabolism. For this, we generated single, double and triple mutants of the three regulators, and analysed their growth and enzyme and gene expression profiles. Our results clearly demonstrated that GalX, GalR and AraR co‐regulate d‐galactose catabolism in *A. nidulans*. GalX has a prominent role on the regulation of genes of d‐galactose oxido‐reductive pathway, while AraR can compensate for the absence of GalR and/or GalX.

## Graphical abstract

This study demonstrates a complex regulation of l‐arabinose and d‐galactose metabolism in the fungus *Aspergillus nidulans*. Roles for at least three transcriptional regulators have been established.

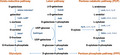

## Introduction

d‐Galactose and l‐arabinose are commonly found together in plant cell wall polysaccharides, such as xylan, xyloglucan, arabinogalactan and rhamnogalacturonan I and II (Fry, 1989; Fitzpatrick *et al*., 2004; Wong, 2008; Caffall and Mohnen, 2009; Kiran *et al*., 2013). In filamentous fungi, the carbon catabolic pathways of these monosaccharides can be active at the same time, enabling simultaneous use of these monosaccharides (Chroumpi *et al*., 2021).

**Fig. 1:**
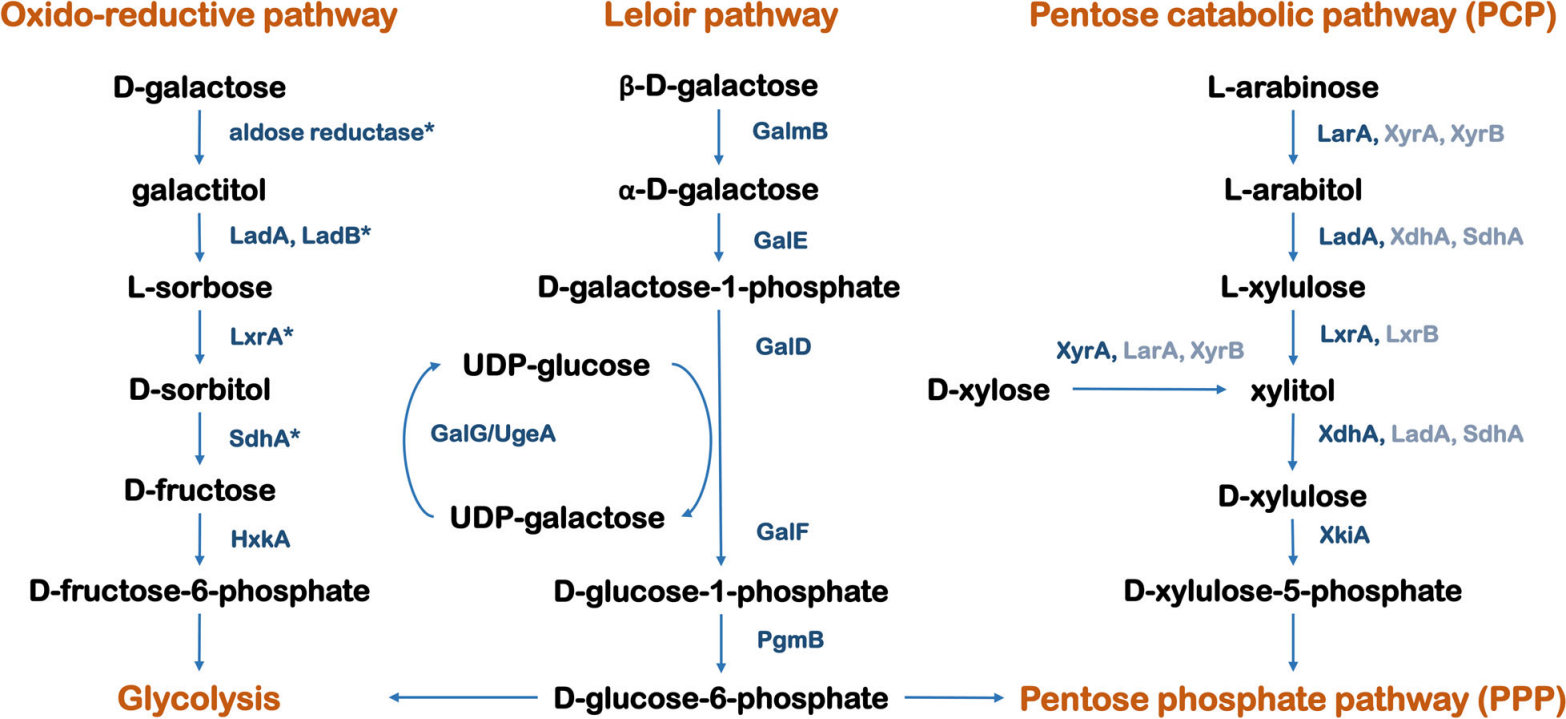
The d‐galactose oxido‐reductive pathway, Leloir pathway and pentose catabolic pathway in *Aspergillus nidulans*. Unidentified or unconfirmed enzymes are marked with a star. Gene function and numbers are: LadA, l‐arabitol dehydrogenase (AN0942); LadB, galactitol dehydrogenase (AN4336); LxrA, LxrB, l‐xylulose reductase (AN10169, AN8819); SdhA, sorbitol dehydrogenase (AN2666); HxkA, hexokinase (AN7459); GalmB, galactose‐1‐epimerase (AN3432); GalE, galactokinase (AN4957); GalD, d‐galactose‐1‐phosphate‐uridylotransferase (AN6182); GalF, UTP‐glucose‐1‐phosphate uridyl transferase (AN9148); GalG/UgeA, UDP‐galactose‐4‐epimerase (AN4727); PgmB, phosphoglucomutase (AN2867); LarA, l‐arabinose reductase (AN7193); XyrA, XyrB, d‐xylose reductase (AN0423, AN1274); XdhA, xylitol dehydrogenase (AN9064); XkiA, d‐xylulose kinase (AN8790).

The key metabolic genes, enzymes and carbon catabolic pathways of *Aspergillus* and other fungi that convert monomers present in plant polysaccharides have been studied for decades (Khosravi *et al*., 2015; Aguilar‐Pontes *et al*., 2018; Chroumpi *et al*., 2020b). The pentoses l‐arabinose and d‐xylose are converted via the pentose catabolic pathway (PCP) through a number of reductase and dehydrogenase catalysed reactions, which was recently updated for *Aspergillus niger* (Fig. 1) (Chroumpi *et al*., 2020b).

There are several d‐galactose catabolic pathways in filamentous fungi. The best‐known pathway is the Leloir pathway, which exists in both prokaryotic and eukaryotic microorganisms (Holden *et al*., 2003). All the enzymes of the Leloir pathway in *A. nidulans* have been described previously (Roberts, 1970). d‐Galactose is phosphorylated to d‐galactose‐1‐phosphate by galactokinase (GalE), and then further converted to UDP‐galactose and d‐glucose‐1‐phosphate by d‐galactose‐1‐phosphate uridylyltransferase (GalD). UDP‐galactose can be converted back to UDP‐glucose by UDP‐galactose 4‐epimerase (GalG) and the conversion of d‐glucose‐1‐phosphate to d‐glucose‐6‐phosphate is catalysed by phosphoglucomutase (PgmB), which eventually enters glycolysis or the PPP (Fekete *et al*., 2008; Flipphi *et al*., 2009; Alam and Kaminskyj, 2013).

An alternative d‐galactose oxido‐reductive pathway, with similarity to the PCP, has been described in *A. nidulans* (Fekete *et al*., 2004), and has also been identified in *Trichoderma reesei* (Pail *et al*., 2004; Seiboth *et al*., 2007) and *A. niger* (Koivistoinen *et al*., 2012; Mojzita *et al*., 2012a, 2012b). In this pathway, d‐galactose is converted to d‐fructose‐6‐phosphate in five enzymatic steps, which then enters glycolysis (Fekete *et al*., 2004). The enzymes in this pathway differ between the three species. Only some enzymes involved in the specific steps have been identified in *A. nidulans,* but there are some similarities to *T. reesei* (Mojzita *et al*., 2012a). Three enzymes of the PCP, Xyl1, Lad1 and Xdh1, are involved in d‐galactose oxido‐reductive pathway in *T. reesei*, and aldose reductase (Xyl1) is a main enzyme in the reduction of d‐galactose to galactitol (Seiboth *et al*., 2007). d‐Xylose reductase (XyrA) has also been suggested to convert d‐galactose to galactitol in *A. niger* (Mojzita *et al*., 2012b), but a recent study disproved that claim (Chroumpi *et al*., unpublished results). Which aldose reductase catalyses this conversion in *A. nidulans* remains unknown. l‐Arabitol dehydrogenase (Lad1) from the PCP catalyses the oxidization of galactitol to l‐xylo‐3‐hexulose in *T. reesei*, but this reaction is catalysed by a specific d‐galactitol dehydrogenase (LadB) instead of LadA in *A. niger*, which is not related to the PCP (Pail *et al*., 2004; Mojzita *et al*., 2012b). The *ladB* ortholog also exists in *A. nidulans* and is likely responsible for this reaction. However, the product of galactitol oxidation was identified as l‐sorbose in *A. nidulans* (Fekete *et al*., 2004). The conversion of l‐sorbose to d‐sorbitol is suggested to be catalysed by l‐xylulose reductase or a similar enzyme (Seiboth and Metz, 2011). Conversely, the reduction of l‐xylo‐hexulose to d‐sorbitol is catalysed by l‐xylo‐3‐hexulose reductase, Lxr4 in *T. reesei* and XhrA in *A. niger* (Mojzita *et al*., 2012a). d‐Sorbitol is suggested to be converted to d‐fructose by sorbitol dehydrogenase (SdhA) in *A. niger*, but a recent study indicated that an alternative sorbitol dehydrogenase may in fact be responsible for this conversion (Chroumpi *et al*., unpublished data). This reaction was described to be catalysed by xylitol dehydrogenase (Xdh1) from the PCP in *T. reesei* (Koivistoinen *et al*., 2012; Mojzita *et al*., 2012a).

Transcriptional regulators control the production of polysaccharide degrading enzymes and enzymes of the carbon catabolic pathways for the released monosaccharides. Two transcriptional regulators, XlnR and AraR, together control the PCP in *Aspergillus* (Battaglia *et al*., 2011a, 2011b). XlnR is induced by d‐xylose, while AraR is induced by l‐arabinose/l‐arabitol (van Peij *et al*., 1998; Battaglia *et al*., 2011b). AraR is the main regulator of the l‐arabinose‐specific PCP genes (LarA, LadA, LxrA), and XlnR regulates *xyrA*. The last two PCP genes (*xdhA*, *xkiA*) are regulated by both regulators (Hasper *et al*., 2000; de Groot *et al*., 2007; Battaglia *et al*., 2011b).

Two other transcriptional regulators, GalX and GalR, control d‐galactose release and catabolism in *A. nidulans* (Christensen *et al*., 2011). GalX is conserved in most Aspergilli, while GalR is unique to section *Nidulantes*, such as *A. nidulans*, *Aspergillus sydowii* and *Aspergillus versicolor* (Kowalczyk *et al*., 2015). In a previous study, the interaction between three transcription factors (XlnR, AraR and GalR) in *A. nidulans* was investigated (Kowalczyk *et al*., 2015), which demonstrated that to a small extent XlnR, but more prominently AraR not only regulate the PCP, but also the oxido‐reductive d‐galactose catabolic pathway. In contrast, GalR only controls the genes of the oxido‐reductive d‐galactose catabolic pathway (Kowalczyk *et al*., 2015). It also suggested that three regulators are not the only regulators involved in the d‐galactose oxido‐reductive pathway and GalX is likely to regulate some enzymes of d‐galactose catabolism directly.

In this study, the possible interaction between GalX, GalR and AraR in d‐galactose and/or l‐arabinose catabolism in *A. nidulans* was investigated in detail. Single, double and triple mutants of the three regulators were generated using CRISPR/Cas9 technology, and their growth, specific enzyme activities, sugar utilization rates and gene expression profiles were analysed.

## Results and discussion

### Extreme low concentrations of l‐arabinose can induce d‐galactose catabolism mediated by AraR

**Fig. 2:**
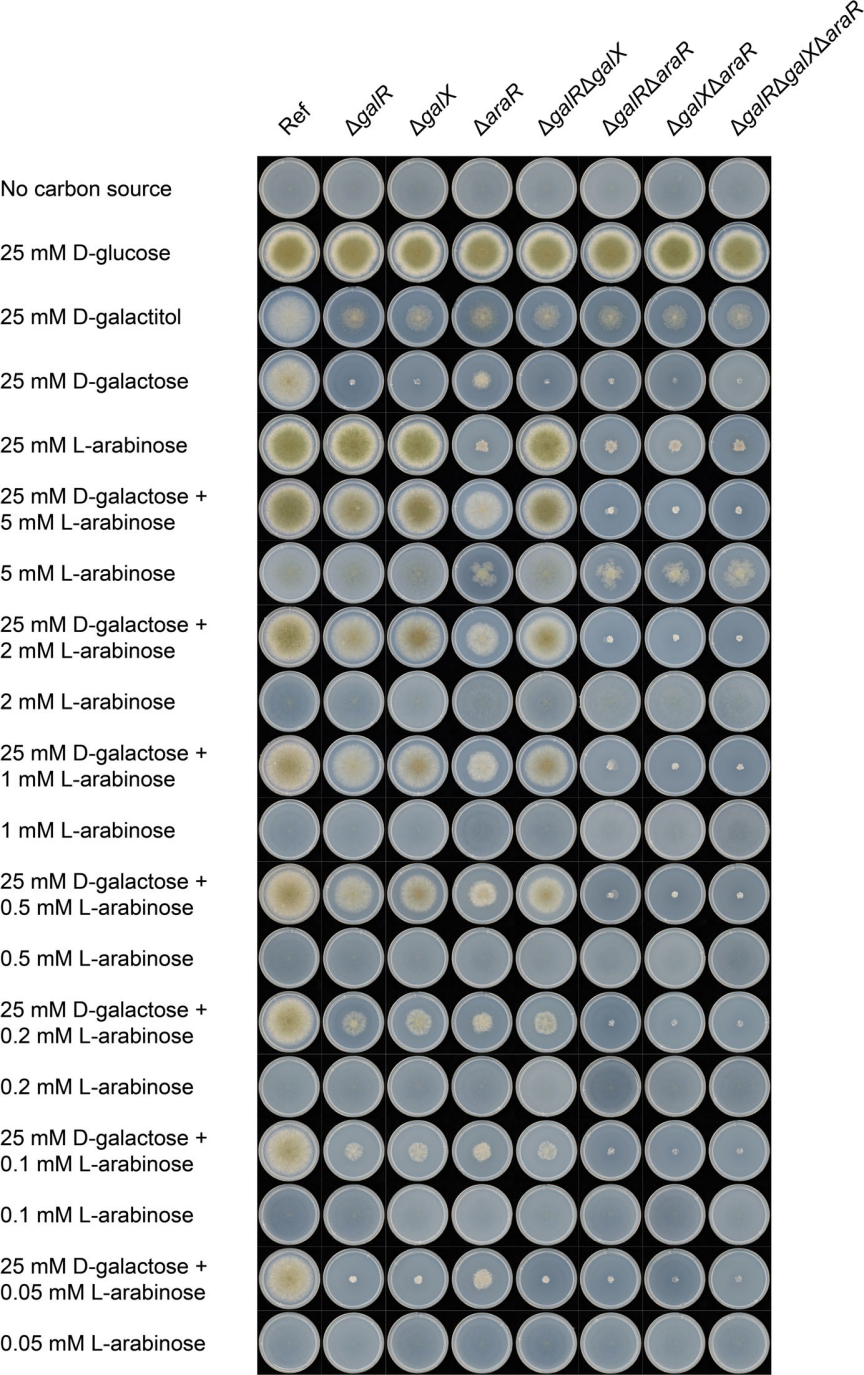
Growth profiling of the *Aspergillus nidulans* reference strain and deletion mutants on different carbon sources.

Single, double and triple deletion mutants of *galR*, *galX* and *araR* were generated to investigate possible interaction between GalX, GalR and AraR in d‐galactose and/or l‐arabinose catabolism in *A. nidulans*. Growth on d‐galactose was almost abolished in all strains in which *galR* and/or *galX* was deleted, while the single deletion of *araR* resulted in significant reduction of growth on d‐galactose (Fig. 2). This indicates that GalR and GalX are the main regulators of d‐galactose catabolism, while AraR has a smaller role. As GalX controls the expression of *galR* (Christensen *et al*., 2011), this puts GalX highest in the hierarchy of regulatory control of d‐galactose catabolism.

Growth of all single mutants on galactitol was highly reduced, while the growth of double and triple mutants did not reduce further (Fig. 2). It indicates GalX, GalR and AraR all regulate galactitol utilization. Galactitol is an intermediate in the d‐galactose oxido‐reductive pathway. In a previous study, growth on this compound was not reduced when *xlnR* was disrupted, so XlnR does not seem to be a predominant regulator of galactitol or d‐galactose utilization (Kowalczyk *et al*., 2015). According to these results, there are supposed to be more regulators (except XlnR, AraR, GalR and GalX) involved in the regulation of galactitol or d‐galactose utilization.

Growth on l‐arabinose was highly reduced when *araR* was deleted (Fig. 2), confirming that AraR regulates l‐arabinose utilization. The residual growth on this sugar can be attributed to the influence of XlnR as it also activates the expression of some genes of the PCP (Kowalczyk *et al*., 2015).

To further study the interaction between GalX, GalR and AraR, we also used the mixtures of d‐galactose and l‐arabinose as carbon sources. Increasing concentrations of l‐arabinose resulted in a gradual growth increase in the single and double mutants of *galR* and *galX* on the mixed carbon sources compared to the sole carbon source. Their growth was comparable to that of the reference strain, especially on the mixture of 25 mM d‐galactose and 5 mM l‐arabinose. These results showed that already at very low concentrations l‐arabinose can induce d‐galactose utilization mediated by AraR.

### d‐Galactose utilization is induced by l‐arabinose mediated by AraR

**Fig. 3:**
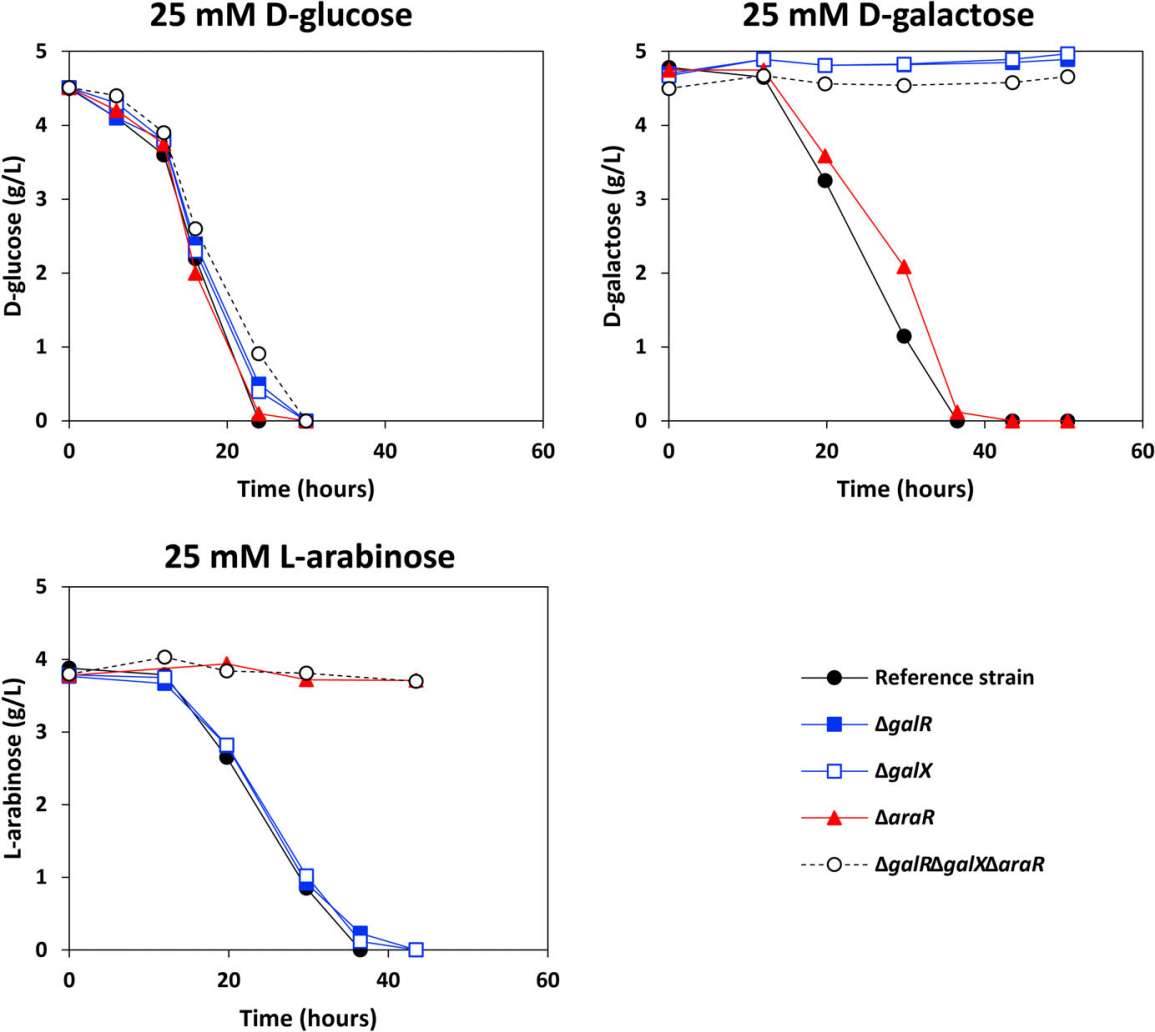
d‐Glucose, d‐galactose and l‐arabinose utilization of the *Aspergillus nidulans* reference strain and mutants.

*Aspergillus nidulans* can consume d‐galactose and l‐arabinose simultaneously, and the utilization rate of l‐arabinose is faster in the presence than in the absence of d‐galactose (Németh *et al*., 2019). In this study, we also determined sugar utilization rates of the reference strain and mutants in liquid culture. First, we assessed the respective utilization rates of d‐glucose, d‐galactose and l‐arabinose in the reference strain, single (Δ*galR*, Δ*galX* and Δ*araR*) and triple (Δ*galR*Δ*galX*Δ*araR*) deletion mutants (Fig. 3 and Table 1). The utilization rates of d‐glucose in all strains were similar as expected. When *galR* and/or *galX* were deleted, the utilization of d‐galactose was completely abolished. The transcriptomic data (Fig. S1) showed that the genes AN4590 and AN9173, encoding two putative major facilitator superfamily (MFS) proteins, were highly expressed on l‐arabinose and d‐galactose compared to d‐glucose, and also significantly downregulated on l‐arabinose when AraR was absent or on d‐galactose when GalR and/or GalX were absent. This could suggest that these genes encode l‐arabinose transporters and may have partial specificity to d‐galactose. Gsx1 (AN9295) is a predicted glucose/xylose–H^+^ symporter (Yang *et al*., 2009) and the expression level of its encoding gene was also significantly downregulated on d‐galactose when *galR* and/or *galX* were deleted. The significantly reduced expression levels of these three genes could be part of the reason why three mutants Δ*galR*, Δ*galX* and Δ*galR*Δ*galX*Δ*araR* lost the ability to utilize d‐galactose. The gene AN2665 encoding another MFS protein was highly expressed on d‐galactose compared to d‐glucose and also downregulated on d‐galactose when *galX* was deleted, suggesting it could be a specific d‐galactose transporter. The identical results for these three strains likely indicate that GalR and GalX control d‐galactose transport directly. The deletion of *araR* slightly affected the utilization of d‐galactose compared to the reference strain. The reason could be that expression levels of transporter genes AN4590, AN9173 and *gsx1* were significantly downregulated when *araR* was deleted on d‐galactose. The utilization of l‐arabinose was completely abolished when *araR* was absent, possibly in part due to the significantly downregulated expression of predicted MFS transporter encoding genes AN1276, AN9173, AN4590, AN8400, AN8467 and *gsx1* on l‐arabinose (Fig. S1). However, the deletion of *galR* or *galX* had no influence on the utilization of l‐arabinose. These results indicate the l‐arabinose transport is fully under control of AraR.

**Table 1:** d‐Glucose, d‐galactose and l‐arabinose utilization rates of the *Aspergillus nidulans* reference strain and mutants.

Strains	**d** ‐Glucose (g l^−1^ h^−1^)	**d** ‐Galactose (g l^−1^ h^−1^)	**l** ‐Arabinose (g l^−1^ h^−1^)
Reference strain	0.3	0.19	0.158
Δ*galR*	0.27	0	0.147
Δ*galX*	0.277	0	0.152
Δ*araR*	0.29	0.182	0
Δ*galR*Δ*galX*Δ*araR*	0.24	0	0

**Fig. 4:**
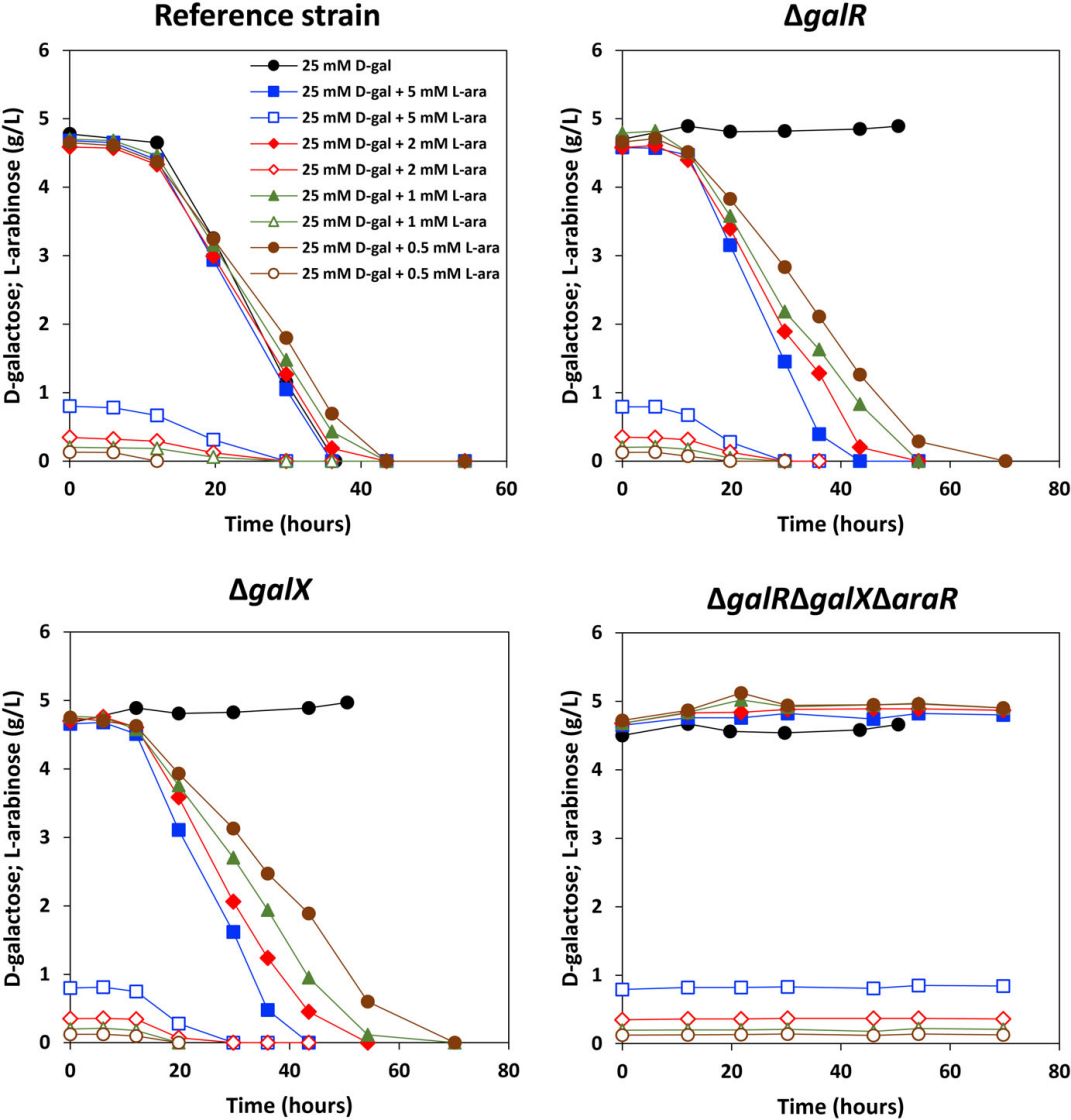
d‐Galactose and l‐arabinose utilization of the *Aspergillus nidulans* reference strain and mutants in the mixtures of these two carbon sources. Open markers: l‐arabinose concentration; closed markers: d‐galactose concentration. d‐gal: d‐galactose; l‐ara: l‐arabinose.

**Fig. 5:**
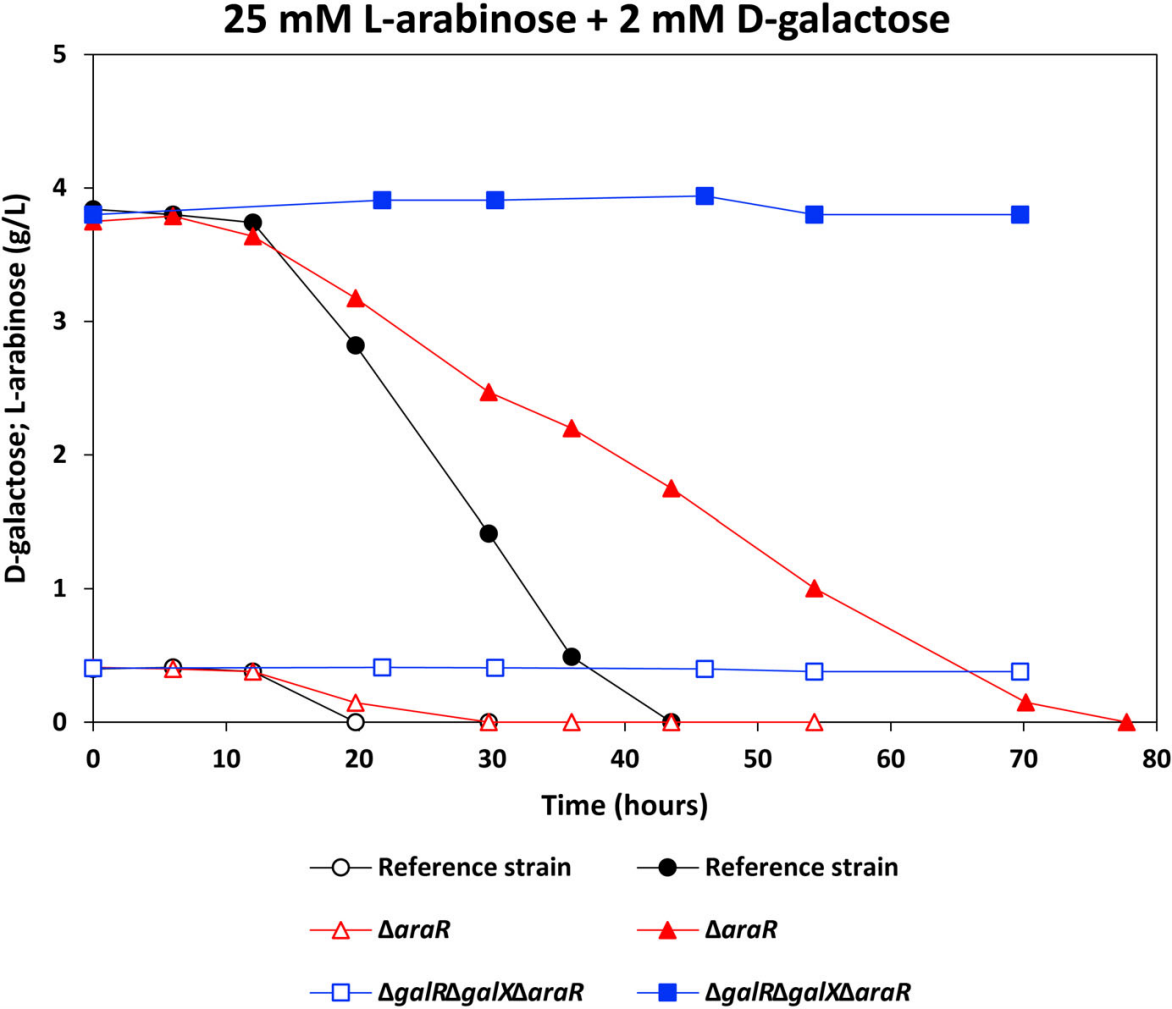
d‐Galactose and l‐arabinose utilization of the *Aspergillus nidulans* reference strain and mutants in the mixture of 25 mM l‐arabinose + 2 mM d‐galactose. Open markers: d‐galactose concentration; closed markers: l‐arabinose concentration.

Moreover, we determined whether the addition of l‐arabinose at different concentrations (0.5, 1, 2 and 5 mM) affected the utilization of d‐galactose (Fig. 4 and Table 2). The utilization rate of d‐galactose in the reference strain was slightly slower when l‐arabinose was present. The presence of l‐arabinose activated the utilization of d‐galactose in Δ*galR* and Δ*galX*, but did not result in d‐galactose utilization when *araR* was deleted (Δ*galR*Δ*galX*Δ*araR*). The expression levels of MFS transporter encoding genes AN4590, AN8467 and AN9173 were highly reduced in Δ*galR*, Δ*galX* and Δ*galR*Δ*galX*Δ*araR* on d‐galactose, but the addition of 5 mM l‐arabinose restored their expression in Δ*galR* and Δ*galX* to the levels in the reference strain, which could contribute to the restoration of d‐galactose utilization in these two mutants (Fig. S1). However, their expression in Δ*galR*Δ*galX*Δ*araR* remained at very low levels when adding 5 mM l‐arabinose to d‐galactose, which could cause the complete stop of d‐galactose utilization in this mutant even after addition of 5 mM l‐arabinose. These results clearly showed that l‐arabinose induction of d‐galactose utilization is mediated by AraR. It is worth noting that the utilization rates of d‐galactose in Δ*galR* and Δ*galX* did not totally restore to the level of the reference strain when l‐arabinose was present. This is consistent with their growth phenotypes on the corresponding carbon sources, showing that in the presence of l‐arabinose, AraR can largely, but not fully compensate for the lack of GalR or GalX in regulating d‐galactose catabolism. Addition of 2 mM d‐galactose to l‐arabinose resulted in l‐arabinose utilization in Δ*araR*, but not in Δ*galR*Δ*galX*Δ*araR*, indicating that GalR and/or GalX can partially restore l‐arabinose utilization in the presence of D‐galactose (Fig. 5 and Table 2).

**Table 2:** d‐Galactose and l‐arabinose utilization rate of the *Aspergillus nidulans* reference strain and mutants in the mixtures of d‐galactose and l‐arabinose.

d‐Galactose utilization rate				
Carbon sources	Reference strain	Δ*galR*	Δ*galX*	Δ*galR*Δ*galX*Δ*araR*
25 mM d‐galactose	0.19	0	0	0
25 mM d‐galactose + 5 mM l‐arabinose	0.184	0.17	0.165	0
25 mM d‐galactose + 2 mM l‐arabinose	0.172	0.133	0.135	0
25 mM d‐galactose + 1 mM l‐arabinose	0.168	0.118	0.109	0
25 mM d‐galactose + 0.5 mM l‐arabinose	0.151	0.102	0.093	0

l‐Arabinose utilization rate			
Carbon sources	Reference strain	Δ*araR*	Δ*galR*Δ*galX*Δ*araR*
25 mM l‐arabinose	0.158	0	0
25 mM l‐arabinose + 2 mM d‐galactose	0.136	0.06	0

### The single deletions of galR, galX and araR have a greater effect on enzymes of the d‐galactose oxido‐reductive pathway than on those of the Leloir pathway

Galactokinase (GalE) catalyses the conversion of d‐galactose to d‐galactose‐1‐phosphate in the Leloir pathway and galactitol dehydrogenase (LadB), instead of l‐arabitol dehydrogenase (LadA), catalyses the conversion of galactitol to l‐sorbose in the oxido‐reductive pathway of *A. nidulans*. In order to study the induction of d‐galactose catabolism by l‐arabinose in *A. nidulans* in detail, we assayed activities of these two catabolic enzymes from pre‐culture (2% d‐fructose) and main culture (25 mM d‐galactose and 5 mM l‐arabinose) in the reference strain and single deletion mutants (Δ*galR*, Δ*galX* and Δ*araR*). In a previous study, the expression of *galE* was reduced to a basal level in Δ*galR* and no expression was observed in Δ*galX* on d‐galactose (Christensen *et al*., 2011). However, there was a basal level of galactokinase activity in the pre‐culture of all *A. nidulans* strains, while this was not the case for l‐arabitol dehydrogenase in this study (Table S1 upper table panel).

In the main culture (Table S1 lower table panel), the galactokinase activity in Δ*araR* was almost the same to the reference strain, while its activity slightly decreased in Δ*galR* and Δ*galX*. However, these decreased values were still higher than the basal levels in the pre‐culture. These results were consistent with the decreased expression level of *galE* in the three single deletion mutants (Δ*galX*, Δ*galR* and Δ*araR*) compared to the reference strain on 25 mM d‐galactose and 5 mM l‐arabinose in this study (Fig. S2). We tested *in vitro* enzyme activity of l‐arabitol dehydrogenase on l‐arabitol and galactitol. When using l‐arabitol as the substrate, enzyme activity of l‐arabitol dehydrogenase decreased in Δ*galR* and conversely increased in Δ*galX* compared to the reference strain. The possible reason of increased enzyme activity on l‐arabitol in Δ*galX* is that the deletion of *galX* slightly increased expression level of *ladA* induced by 5 mM l‐arabinose according to the transcriptome data (Fig. S2). The decreased enzyme activity on l‐arabitol in Δ*galR* could be explained by the slightly reduced expression level of *xdhA*, rather than *ladA*, compared to the reference strain. However, the enzyme activity decreased in Δ*galR* and Δ*galX* on galactitol, especially in Δ*galX*, which is consistent with the significantly reduced expression level of *ladB* in both mutants compared to the reference strain (Fig. S2). The deletion of *araR* decreased both specific activities by 28–44% compared to the reference strain, which can be the reason of the reduced growth of Δ*araR* on galactitol. The decreased expression level of *ladA* in Δ*araR* can explain the reduced enzyme activity of l‐arabitol dehydrogenase on l‐arabitol. However, the expression level of *ladB* increased slightly in Δ*araR* compared to the reference strain, which contradicts with the reduced enzyme activity of l‐arabitol dehydrogenase on galactitol (Fig. S2). In a previous study, LadB and LadA from *A. niger* have similar *in vitro* activity with galactitol and LadA induced on d‐xylose could substitute for LadB (Mojzita *et al*., 2012b). Therefore, the decreased expression level of *ladA* in Δ*araR* could be a possible reason for the reduced enzyme activity on galactitol. These results could also explain the slightly lower d‐galactose utilization rates (Table 2) and poorer growth of Δ*galR* and Δ*galX* (Fig. 2) compared to the reference strain on 25 mM d‐galactose and 5 mM l‐arabinose.

### GalX, GalR and AraR all regulate d‐galactose catabolism, but GalX has the biggest impact

**Fig. 6:**
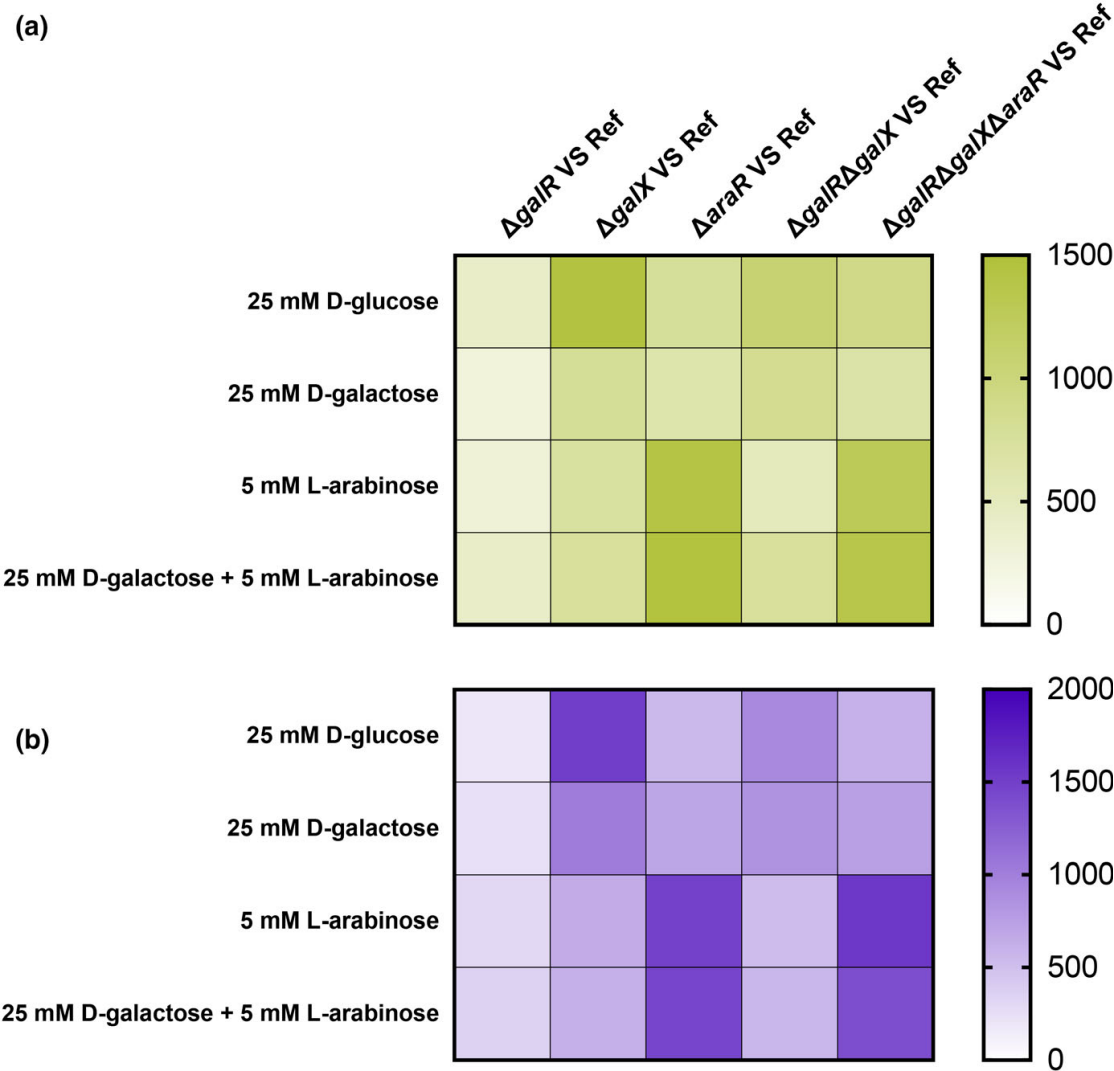
The number of differentially expressed genes in *Aspergillus nidulans* mutants compared to the reference strain on different carbon sources. Highly (A) upregulated and (B) downregulated genes in mutants. Transcripts were considered as differentially expressed if the DESeq2 fold change was > 2 and *P*_adj_ < 0.01.

According to growth phenotypes and sugar utilization rates of the reference strain and mutants, GalX, GalR and AraR are all involved in d‐galactose catabolism. The analysis of transcriptome data was performed to study transcriptional changes between the reference strain and mutants. The number of differentially expressed genes (DEGs, Fig. 6) showed that the single deletion of *galX* or *araR* has a wider influence on gene expression than the deletion of *galR*, indicating broader regulatory functions of *galX* and *araR* under these conditions. Surprisingly, there was a large number of DEGs due to *galX* deletion on d‐glucose, even more than on d‐galactose, suggesting that GalX may have an additional role in d‐glucose catabolism through glycolysis. These include some GalX‐regulated glycolytic genes on d‐glucose, such as *pgkA*, *pgmA*, *pfkA* and *glkA*. The absence of *araR* affected the expression of many genes on l‐arabinose with and without d‐galactose, confirming its clear role in l‐arabinose utilization.

The significantly decreased expression levels of the three regulators in the corresponding mutants confirmed their deletion (Fig. S2). The expression level of *galR* also reduced significantly in Δ*galX*, confirming that GalX controls GalR (Christensen *et al*., 2011). It is worth noting that the expression level of *xlnR* was highly increased compared to the reference strain when *araR* was absent on l‐arabinose, suggesting that XlnR might be able to partially compensate for the absence of AraR.

PCP genes *larA*, *ladA*, *lxrA*, *xdhA* and *xkiA* were highly expressed in the reference strain on l‐arabinose, and were expressed at very low levels when *araR* is deleted on l‐arabinose (Fig. S2). The decreased expression levels of the first three genes in PCP (*larA*, *ladA* and *lxrA*) explains the reduced growth of Δ*araR* on l‐arabinose. The PCP gene *xyrA* was highly expressed on l‐arabinose, but not downregulated when *araR* was deleted, indicating that this gene is mainly controlled by XlnR as mentioned in the previous study (Kowalczyk *et al*., 2015). Most of the PCP genes seem to be induced on d‐galactose compared to d‐glucose, but show lower expression levels on d‐galactose than on l‐arabinose. As the deletion of *galR* and/or *galX* did highly reduce the expression of *lxrA* and *xkiA* on a mixture of d‐galactose and l‐arabinose, we cannot exclude the involvement of GalR and/or GalX in regulation of PCP genes under these conditions.

All genes involved in Leloir pathway were expressed on d‐galactose and their expression levels were not affected by the deletion of the three regulators (Fig. S2). Not all the genes of d‐galactose oxido‐reductive pathway have been identified in *A. nidulans*. The first enzyme, aldose reductase, involved in the conversion of d‐galactose to galactitol remains unknown. The *ladB* (AN4336) gene, which encodes a specific galactitol dehydrogenase (LadB) in *A. niger*, also exists in *A. nidulans* and is likely responsible for this conversion of galactitol to l‐sorbose (Pail *et al*., 2004; Mojzita *et al*., 2012b). The highly reduced growth of Δ*galX*, double and triple mutants on galactitol, but not abolished, indicated that other enzymes could be involved in this conversion, as the expression of *ladB* was highly decreased to around zero. The expression of *ladB* was almost lost when *galX* was deleted confirming that GalX regulates this gene (Christensen *et al*., 2011). In this study, the expression of *ladA* was induced on d‐galactose, but much lower than on l‐arabinose, and was not affected when three regulators were deleted on d‐galactose. Therefore, our results confirmed the existence of other enzymes of this reaction. The slightly decreased expression of *ladB* can explain the decreased growth of Δ*galR* on galactitol.

A previous study determined that a putative reductase encoding gene *red1* (AN7914) was co‐regulated by XlnR, GalR and AraR in *A. nidulans* and was a strong candidate as the unconfirmed l‐sorbose reductase (Kowalczyk *et al*., 2015). However, the expression of *red1* was not highly induced in the reference strain and did not reduce in any of the mutants on d‐galactose in our study, so our results could not support its function in this conversion (Fig. S2). The expression of *lxrA* (AN10169) was induced on d‐galactose, but much lower than on l‐arabinose. The expression level of *lxrA* was decreased in all mutants by up to 86% compared to the reference strain on d‐galactose, indicating that the expression of *lxrA* is regulated by GalX, GalR and AraR. In *T. reesei*, the l‐xylulose reductase (LXR1) has activity with l‐xylulose, d‐xylulose, d‐fructose and L‐sorbose (Richard *et al*., 2002). Therefore, LxrA may be responsible for the conversion of l‐sorbose to d‐sorbitol, at least partially. Its function and the involvement of other enzymes in d‐galactose oxido‐reductive pathway require further studies. The decreased expression level of *lxrA* can be a reason of the reduced growth of all mutants on d‐galactose and galactitol.

d‐Sorbitol is converted to d‐fructose by sorbitol dehydrogenase (SdhA) in *A. niger* (Koivistoinen *et al*., 2012). The expression of gene AN2666, the ortholog of *sdhA* in *A. nidulans*, was highly induced in the reference strain and its expression level slightly reduced in all mutants on d‐galactose, but significantly reduced in Δ*galX*, indicating the expression of *sdhA* is regulated by GalX, GalR and AraR (Fig. S2). The single deletion of *galX* had the strongest effect on expression of *sdhA*, but the expression of this gene was not abolished in the triple deletion mutant on d‐galactose. The residual expression of *sdhA* could be caused by the regulation of XlnR because a previous study demonstrated that this gene is also regulated by XlnR (Kowalczyk *et al*., 2015). These results confirmed that both *ladB* and *sdhA* were highly induced by d‐galactose and regulated by different regulators, so they are involved in d‐galactose oxido‐reductive pathway. Our results also confirmed that GalX directly regulates these two enzymes of d‐galactose catabolism in *A. nidulans*. The last step of d‐galactose oxido‐reductive pathway is catalysed by hexokinase. The expression of *hxkA* (AN7459) was not significantly affected in all mutants, showing a constitutive level of expression. Growth was not abolished on d‐galactose when *galR* and/or *galX* were absent (Fig. 2). The unaffected Leloir pathway can explain the residual growth of these mutants on d‐galactose and d‐galactose oxido‐reductive pathway can be the preferred pathway in *A. nidulans* as previously proposed (Kowalczyk *et al*., 2015).

## Conclusions

In summary, the results showed that the regulation of d‐galactose catabolism is highly complex and that at least four regulators are involved in its regulation (GalR, GalX, XlnR, AraR). The growth phenotypes and sugar utilization on the mixture of d‐galactose and l‐arabinose demonstrated a clear role for AraR in d‐galactose utilization. The transcriptome data indicated that GalX has a wider effect on regulation of genes involved in d‐galactose and d‐glucose catabolism than GalR and AraR, while the regulatory function of GalR is not notable on the expression of catabolic genes. It may therefore indicate that GalR mainly regulates d‐galactose transport based on sugar utilization study. Several enzymes in d‐galactose oxido‐reductive pathway remain to be confirmed, and studying these enzymes may be able to provide more detailed evidences about the regulatory role of GalR. The compensation phenomenon between different regulators was also confirmed in this study as previously described, which can ensure the fungus quickly adapt constantly changing environment.

## Experimental procedures

### Strains, media and culture conditions

*Escherichia coli* DH5α was used for plasmid construction and was grown on Luria–Bertani (LB) medium supplemented with 50 μg ml^−1^ ampicillin. *Aspergillus nidulans* strains used in this study were deposited at the CBS culture collection of the Westerdijk Fungal Biodiversity Institute with numbers shown in Table S2. The reference strain *A. nidulans* FGSC A1149 is a uracil auxotrophic and *nkuA* deletion strain used as a parental strain for transformation and efficient gene knockouts. *Aspergillus nidulans* strains were grown at 37°C on complete medium (CM) or minimal medium (MM) supplemented with required carbon source (de Vries *et al*., 2004). Solid media were amended with 1.5% (w/v) agar. Uridine (1.22 g l^−1^) and pyridoxine (1 mg l^−1^) were supplemented for all auxotrophic strains, and 1.3 mg ml^−1^ 5‐fluoroorotic acid (5‐FOA) was added in the solid medium for counter selecting colonies containing the *pyrG* marker gene on ANEp8‐Cas9 plasmids.

*Aspergillus nidulans* strains were grown on CM plates with 1% d‐glucose at 37°C for 5 days. Spores were harvested in ACES buffer and were counted using a haemocytometer. Solid MM was used for growth profiles supplemented with different monosaccharides, including 25 mM d‐glucose, 25 mM galactitol, 25 mM d‐galactose, 25 mM l‐arabinose, 5 mM l‐arabinose, 2 mM l‐arabinose, 1 mM l‐arabinose, 0.5 mM l‐arabinose, 0.2 mM l‐arabinose, 0.1 mM l‐arabinose and 0.05 mM l‐arabinose as well as mixtures of l‐arabinose with these concentrations and 25 mM d‐galactose. Two hundred spores in 5 μL ACES buffer were inoculated on the plates and incubated at 37°C for 5 days.

### Construction of deletion mutants

The CRISPR/Cas9 system used for the construction of deletion mutants was described previously (Song *et al*., 2018). The gRNA sequences of all ANEp8‐Cas9 plasmids were identified using Geneious R11 software (https://www.geneious.com) based on *A. niger* NRRL3 genome. The gene deletion cassettes were constructed by fusion of upstream and downstream DNA fragments in a PCR using Phusion^TM^ High‐Fidelity DNA Polymerase (Thermo Fisher Scientific, Nieuwegein, The Netherlands). Upstream and downstream DNA fragments were amplified using genomic DNA of *A. nidulans* FGSC A1149 as template. A barcode sequence was used as overlapping region for the fusion of two fragments. The primers used in this study are listed in Table S3.

*Aspergillus nidulans* protoplasting and transformation were performed as previously described (Kun *et al*., 2020). Each transformation required 1 μg ANEp8‐Cas9 plasmid and 5 μg corresponding gene deletion cassette. Several colonies from transformation plates were selected and purified by single colony streaking on MM plates twice. Colonies were subsequently re‐cultivated twice on MM plates with uridine to remove the self‐replicating ANEp8‐Cas9 plasmid. Genomic DNA of putative mutants was isolated and used as a template of colony PCR. Correct mutants were confirmed by amplifying the target region flanking the Cas9 cut site using primers listed in Table S3. All mutants lacking ANEp8‐Cas9 plasmid were screened by growth on MM plates containing 5‐fluoroorotic acid (5‐FOA) before deposit to the CBS culture collection.

### Transfer experiment, RNA isolation and transcriptome analysis

The transfer experiment was performed in biological triplicate. For pre‐cultures, 10^6^ spores ml^−1^ were inoculated to 250 ml CM with 2% d‐fructose in 1 L Erlenmeyer flasks and incubated in rotary shakers at 37°C, 250 rpm, for 16–18 hours. The mycelia were harvested by filtration on cheesecloth under sterile conditions and washed with MM. Equal amount of mycelia was transferred to 50 ml MM in 250 ml Erlenmeyer flasks supplemented with 25 mM d‐glucose, 25 mM d‐galactose, 5 mM l‐arabinose and a mixture of 25 mM d‐galactose and 5 mM l‐arabinose, and were incubated in rotary shakers at 37°C, 250 rpm. After 2 hours incubation, mycelia were harvested by vacuum filtration, dried between tissue paper and frozen in liquid nitrogen. All samples were stored at −80°C for RNA isolation.

The transcriptomes of all strains were analysed by RNA‐seq. Total RNA was extracted from ground mycelia using TRIzol reagent (Invitrogen, Merelbeke, Belgium) and NucleoSpin RNA Clean‐up Kit (Macherey‐Nagel, Düren, Germany). The quality and quantity of RNA samples were analysed by a RNA6000 Nano Assay using the Agilent 2100 Bioanalyzer (Agilent Technologies, Middelburg, The Netherlands). Purification of mRNA, synthesis of cDNA library and sequencing were conducted at DOE Joint Genome Institute (JGI) as described previously (Chroumpi *et al*., 2020a). Briefly, RNA sample preparation was performed using the Illumina TruSeq Stranded preparation kit and following Illumina poly‐A selection protocol. The prepared libraries were quantified using qPCR and then sequenced on the Illumina NovaSeq sequencer following a 2 × 150 indexed run recipe.

Using BBDuk (https://sourceforge.net/projects/bbmap), raw reads were evaluated for artefact sequence by kmer matching (kmer=25), allowing one mismatch and detected artefact was trimmed from the 3′ end of the reads. RNA spike‐in reads, PhiX reads and reads containing any Ns were removed. Quality trimming was performed using the phred trimming method set at Q6. Finally, following trimming, reads under the length threshold were removed (minimum length 25 bases or one third of the original read length – whichever was longer). Filtered reads from each library were aligned to the *A. niger* NRRL3 (http://genome.jgi.doe.gov/Aspni_NRRL3_1) genome using HISAT2 version 2.1.0 (Kim *et al*., 2015). FeatureCounts (Liao *et al*., 2014) was used to generate the raw gene counts using gff3 annotations. Only primary hits assigned to the reverse strand were included in the raw gene counts (‐s 2 ‐p –primary options). The reads from all RNAseq samples were deposited at the Sequence Read Archive NCBI with sample accession numbers SRP296258–SRP296269, SRP296271–SRP296281, SRP296282–SRP296292, SRP307787–SRP307798, SRP307809–SRP307820 and SRP307825–SRP307836.

Statistical analysis was performed using DESeq2 (Love *et al*., 2014). Transcripts were considered as differentially expressed if the DESeq2 fold change was > 2 and *P*_adj_ < 0.01. Two heat maps were drawn using GraphPad Prism (https://www.graphpad.com/).

### Enzyme activity assays

Pre‐cultures were inoculated with 10^6^ spores ml^−1^ and were grown for 16 hours with CM containing 2% d‐fructose, 1.22 g l^−1^ uridine and 1 mg l^−1^ pyridoxine. Cultures were incubated at 37°C in 500 ml Erlenmeyer flasks containing 100 ml aliquots in a rotary shaker at 200 rpm. Samples were taken right before mycelial transfer. Mycelia were then harvested by filtration on a sintered glass funnel without suction, washed with MM without carbon source and transferred into fresh MM with 25 mM d‐galactose + 5 mM l‐arabinose, supplemented with 1.22 g l^−1^ uridine and 1 mg l^−1^ pyridoxine (referred to as main culture). Samples were taken after 4 hours of incubation to assess induction ability. Preliminary trails had established that 4 hours of contact is the time lapse in which maximal induced enzyme activity levels were achieved, with a minimal variation in the biomass concentration. By that time, both l‐arabinose and d‐galactose have been started to be taken up simultaneously.

To obtain a cell‐free extract, 10 ml of culture broth was withdrawn and suction filtered, and then the harvested mycelia was thoroughly washed with the corresponding buffer used for the respective enzyme activity measurements. The biomass was resuspended in 5 ml of the same buffer, and homogenized in a pre‐cooled Potter‐Elvehjem glass homogenizer. The homogenate was centrifuged at 20 000 x *g* (20 min, 4°C), and the supernatant immediately used to assay the respective enzyme activities.

Galactokinase activity assay was based on the detection of galactose‐1‐phosphate in 1 ml of a reaction mixture containing 10 mM ATP, 20 mM d‐galactose, 10 mM MgSO_4_ and 0.7 ml crude extract in a 0.1‐M phosphate buffer, pH 7.6. The assay was performed at 37°C. The reaction was initiated by the addition of d‐galactose, allowed to proceed for 30 min and then terminated by chilling the mixture on ice. Sulphate was removed by precipitation with an equimolar amount of Ba(OH)_2_, followed by centrifugation (room temperature, 20 000 *g*, 20 min). The supernatant was assayed for the presence of galactose‐1‐phosphate by HPLC using an H^+^ exchange column (Bio‐Rad) at 30°C with 25 mM H_2_SO_4_ as mobile phase with isocratic elution and a refractive index detection. Within the time and conditions of the assay, the formation of galactose‐1‐phosphate was linear with respect to time.

l‐Arabitol dehydrogenase activity assay was performed as described previously (de Vries *et al*., 1994). The respective l‐arabitol and galactitol concentrations in the assay were 100 mM. The reaction was allowed to proceed for 1 h at 37°C, and was then terminated by boiling the mixture for 5 min. The reaction mixture was centrifuged (room temperature, 20 000 *g*, 20 min) and the supernatant used for HPLC analysis.

Specific enzyme activity values are related to mg protein, which was determined by means of a modified Lowry method (Peterson, 1983), using BSA for calibration.

### Sugar utilization rates

The concentration of d‐glucose, d‐galactose and l‐arabinose in the culture broth was determined by HPLC analysis, using an H^+^ exchange column (Bio‐Rad Aminex HPX‐H^+^; Hercules, CA, USA), employing 10 mM H_2_SO_4_ at 55°C as mobile phase. Compounds were detected by means of a refractive index detector (Fekete *et al*., 2002). Each point is the result of two averaged measurements, which deviated by not more than 5%.

## Conflict of interest

The authors declare no competing interests.

## Author contributions

J.M. performed experiments, analysed data and wrote the original manuscript. Z.N. performed experiments and analysed data. M.P., A.L., V.N., E.S., Y.Z. and I.V.G. performed transcriptomic analysis. S.G. supervised part of the research. E.F. and L.K. designed experiments and supervised part of the research. M.R.M. and R.P.dV. designed the experiments, supervised the overall research and reviewed and edited the manuscript. All authors read and approved the manuscript.

## Supporting information


**Fig. S1**. Expression profiles of genes encoding putative MFS transporters in the reference strain and mutants on different carbon sources. The colour code represents averaged and logged expression values (FPKM + 1) of triplicates. glc = 25 mM d‐glucose, gal = 25 mM d‐galactose, ara = 5 mM l‐arabinose, gal+ara = 25 mM d‐galactose + 5 mM l‐arabinose

**Fig. S2**. Expression profiles of genes encoding related transcription factors and enzymes involved in the PCP and d‐galactose catabolic pathway in the reference strain and mutants on different carbon sources. The colour code represents averaged and logged expression values (FPKM + 1) of triplicates. glc = 25 mM d‐glucose, gal = 25 mM d‐galactose, ara = 5 mM l‐arabinose, gal+ara = 25 mM d‐galactose + 5 mM l‐arabinose

**Table S1**. Specific enzyme activities ($U\;{\text{mg}}_{\text{protein}}^{‐1}$) detected from mycelial extracts of *Aspergillus nidulans* strains. Upper table panel: pre‐culture. Lower table panel: main culture.
**Table S2**. *Aspergillus nidulans* strains used in this study.
**Table S3**. Primers used in this study. The guide RNAs (gRNA) for gene deletion are marked in red and the linkers are shown in lowercase.
