# Supplementary material for: GalR, GalX and AraR co‐regulate d‐galactose and l‐arabinose utilization in Aspergillus nidulans
Source: Microb Biotechnol. 2022 Feb 25;15(6):1839–51. doi: 10.1111/1751-7915.14025 (PMC9151342; doi:10.1111/1751-7915.14025)
Supplement: Supplementary file 1 — Fig. S1. Expression profiles of genes encoding putative MFS transporters in the reference strain and mutants on different carbon sources. The colour code represents averaged and logged expression values (FPKM + 1) of triplicates. glc = 25 mM d‐glucose, gal = 25 mM d‐galactose, ara = 5 mM l‐arabinose, gal+ara = 25 mM d‐galactose + 5 mM l‐arabinose [file MBT2-15-1839-s003.docx]

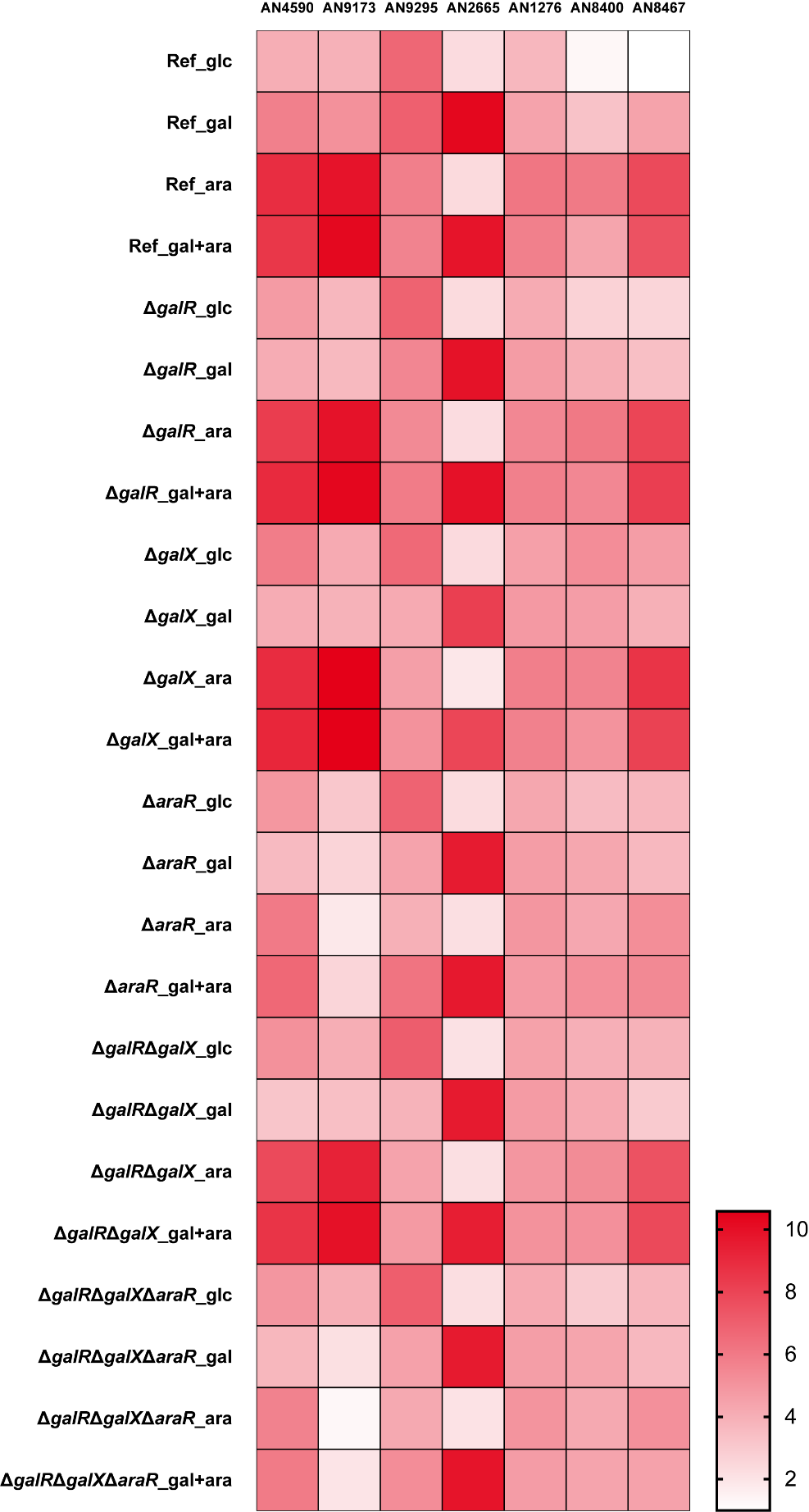


Supplemental Figure S1. Expression profiles of genes encoding putative MFS transporters in the reference strain and mutants on different carbon sources. The color code represents averaged and logged expression values (FPKM + 1) of triplicates. glc = 25 mM D-glucose, gal = 25 mM D-galactose, ara = 5 mM L-arabinose, gal+ara = 25 mM D-galactose + 5 mM L-arabinose.
