# Supplementary material for: GalR, GalX and AraR co‐regulate d‐galactose and l‐arabinose utilization in Aspergillus nidulans
Source: Microb Biotechnol. 2022 Feb 25;15(6):1839–51. doi: 10.1111/1751-7915.14025 (PMC9151342; doi:10.1111/1751-7915.14025)
Supplement: Supplementary file 3 — Table S1. Specific enzyme activities (Umgprotein‐1) detected from mycelial extracts of Aspergillus nidulans strains. Upper table panel: pre‐culture. Lower table panel: main culture. Table S2. Aspergillus nidulans strains used in this study. Table S3. Primers used in this study. The guide RNAs (gRNA) for gene deletion are marked in red and the linkers are shown in lowercase. [file MBT2-15-1839-s001.docx]

**Supplemental Table S1.** Specific enzyme activities (U mg_protein_^-1^) detected from mycelial extracts of *A. nidulans* strains. Upper table panel: pre-culture. Lower table panel: main culture.

|  | Galactokinase | L-arabitol dehydrogenase | |
| --- | --- | --- | --- |
| Assayed *in vitro* substrates | D-galactose | L-arabitol | D-galactitol |
| Reference strain | 0.18 ± 0.02 | < 0.04 | < 0.05 |
| Δ*galR* | 0.13 ± 0.02 | < 0.04 | < 0.05 |
| Δ*galX* | 0.12 ± 0.02 | < 0.04 | < 0.05 |
| Δ*araR* | 0.12 ± 0.02 | < 0.04 | < 0.05 |

|  | Galactokinase | L-arabitol dehydrogenase | |
| --- | --- | --- | --- |
| Assayed *in vitro* substrates | D-galactose | L-arabitol | D-galactitol |
| Reference strain | 0.32 ± 0.04 | 0.38 ± 0.04 | 0.21 ± 0.02 |
| Δ*galR* | 0.22 ± 0.04 | 0.28 ± 0.04 | 0.17 ± 0.01 |
| Δ*galX* | 0.24 ± 0.06 | 0.48 ± 0.04 | 0.06 ± 0.01 |
| Δ*araR* | 0.30 ± 0.03 | 0.21 ± 0.04 | 0.15 ± 0.03 |

**Supplemental Table S2.** *Aspergillus nidulans* strains used in this study.

| Strains | CBS number | Genotype | Reference |
| --- | --- | --- | --- |
| FGSC A1149 (TN02A3) | - | *pyrG89*; *pyroA4*; *nkuA*::*argB* | Nayak *et al.*, 2006 |
| Δ*galR* | CBS 147596 | *pyrG89*; *pyroA4*; *nkuA*::*argB*; Δ*galR* | This study |
| Δ*galX* | CBS 147597 | *pyrG89*; *pyroA4*; *nkuA*::*argB*; Δ*galX* | This study |
| Δ*araR* | CBS 147600 | *pyrG89*; *pyroA4*; *nkuA*::*argB*; Δ*araR* | This study |
| Δ*galR*Δ*galX* | CBS 147598 | *pyrG89*; *pyroA4*; *nkuA*::*argB*; Δ*galR*; Δ*galX* | This study |
| Δ*galR*Δ*araR* | CBS 147601 | *pyrG89*; *pyroA4*; *nkuA*::*argB*; Δ*galR*; Δ*araR* | This study |
| Δ*galX*Δ*araR* | CBS 147602 | *pyrG89*; *pyroA4*; *nkuA*::*argB*; Δ*galX*; Δ*araR* | This study |
| Δ*galR*Δ*galX*Δ*araR* | CBS 147599 | *pyrG89*; *pyroA4*; *nkuA*::*argB*; Δ*araR*; Δ*galR*; Δ*galX* | This study |

**Supplemental Table S3.** Primers used in this study. The guide RNAs (gRNA) for gene deletion are marked in red and the linkers are shown in lowercase.

| Primers | Sequences | Description |
| --- | --- | --- |
| P1 | CAACCTCCAATCCAATTTGACTCCGCCGAACGTACTG | For constructing gRNA |
| P2 | ACTACTCTACCACTATTTGAAAAGCAAAAAAGGAAGGTACAAAAAAGC | For constructing gRNA |
| P3-galR | CGCGTGAACTCGCAACGCGTGACGAGCTTACTCGTTTCG | For constructing gRNA of *galR* |
| P4-galR | ACGCGTTGCGAGTTCACGCGGTTTTAGAGCTAGAAATAGCAAG | For constructing gRNA of *galR* |
| P3-galX | TGGGATATCGAACTTCGGCTGACGAGCTTACTCGTTTCG | For constructing gRNA of *galX* |
| P4-galX | AGCCGAAGTTCGATATCCCAGTTTTAGAGCTAGAAATAGCAAG | For constructing gRNA of *galX* |
| P3-araR | TTGCGAAGTCCCAGCGCTGTGACGAGCTTACTCGTTTCG | For constructing gRNA of *araR* |
| P4-araR | ACAGCGCTGGGACTTCGCAAGTTTTAGAGCTAGAAATAGCAAG | For constructing gRNA of *araR* |
| galR 5' Fw | GCAGGCAGGCGGATTAAAGG | For amplifying *galR* 5’ flank and identifying correct deletion mutation |
| galR 5' Rv | cgatagcgaatcctagcagtAGAGGATGCTCGATGTATTAGG | For amplifying *galR* 5’ flank |
| galR 3' Fw | actgctaggattcgctatcgGCAATAGCTTCTTGAATAGCACC | For amplifying *galR* 3’ flank |
| galR 3' Rv | GTTTACATTCGTCGCAGGCG | For amplifying *galR* 3’ flank and identifying correct deletion mutation |
| galR 5' NEST Fw | TGCGGATATAGTTAGTTGTGG | For fusion of *galR* 5’ and 3’ flanks |
| galR 3' NEST Rv | GCAAACCAAGGACGTTACTG | For fusion of *galR* 5’ and 3’ flanks |
| galX 5' Fw | CGGGTATCTCAAGGGCTTC | For amplifying *galX* 5’ flank and identifying correct deletion mutation |
| galX 5' Rv | cgatagcgaatcctagcagtGCAAACCAAGGACGTTACTG | For amplifying *galX* 5’ flank |
| galX 3' Fw | actgctaggattcgctatcgGTCTTATGGTTATAGATATGC | For amplifying *galX* 3’ flank |
| galX 3' Rv | CAGCGACGTGGTTATCCG | For amplifying *galX* 3’ flank and identifying correct deletion mutation |
| galX 5' NEST Fw | GCAATAGCTTCTTGAATAGCACC | For fusion of *galX* 5’ and 3’ flanks |
| galX 3' NEST Rv | GGATCGAACAGAAGAGGAC | For fusion of *galX* 5’ and 3’ flanks |
| galR::galX 5' NEST Fw | CGCTCTACAGCCAATAGAG | For fusion of *galR*::*galX* 5’ and 3’ flanks with primer galX 3' NEST Rv |
| araR 5' Fw | GGACGCATACGAACGCTG | For amplifying *araR* 5’ flank and identifying correct deletion mutation |
| araR 5' Rv | cgatagcgaatcctagcagtCAGGACGGCATTGGCAAG | For amplifying *araR* 5’ flank |
| araR 3' Fw | actgctaggattcgctatcgGCTCTTCTAATTTGCGTC | For amplifying *araR* 3’ flank |
| araR 3' Rv | CCTATGGGTTGAGTGTGGCAG | For amplifying *araR* 3’ flank and identifying correct deletion mutation |
| araR NEST 5' Fw | GAAGGATGCCTGGTTATCG | For fusion of *araR* 5’ and 3’ flanks |
| araR NEST 3' Rv | CGGATTCTGGAGATTCTTG | For fusion of *araR* 5’ and 3’ flanks |
